# How teacher support, peer connection, and embodied meaning-making interact in drama-based social-emotional learning in higher education

**DOI:** 10.3389/fpsyg.2026.1963943

**Published:** 2026-09-29

**Authors:** Kunqi Wang, Xiyu Dai

**Affiliations:** 1 School of Art and Design, Zhongyuan University of Technology, Zhengzhou, China; 2 Normal School, Changzhou Institute of Technology, Changzhou, China

**Keywords:** basic psychological needs, drama-based social-emotional learning, embodied cognition, higher education, peer support, teacher support

## Abstract

Drama-based social-emotional learning has gained increasing attention in higher education, yet participation in embodied activities does not in itself ensure sustained engagement or social-emotional development. Less is known about how teacher support, peer interaction, and embodied participation become interconnected in classroom practice, or about the conditions under which this process may be disrupted. This multisite interpretive qualitative study was conducted across four universities in eastern China. Data were generated through interviews with 24 students and eight teaching staff, supplemented by 24 reflective journals, 24 classroom observations, and 16 course documents. Reflexive thematic analysis produced three themes: participatory safety and bounded autonomy supported by teacher scaffolding; peer connection and group belonging developed through embodied collaboration; and emotional resonance and self-awareness emerging through embodied meaning-making. Safety, responsiveness, and a sense of meaning were associated with students’ willingness to participate, whereas unclear boundaries, limited peer responsiveness, and excessive emotional intensity were associated with reduced participation. The findings conceptualize drama-based social-emotional learning as interconnected classroom processes involving teacher scaffolding, peer connection, embodied participation, and reflective integration.

## 1 Introduction

In recent years, social-emotional learning has begun to enter higher education curricula, while drama, role-play, scenario-based simulations, and improvisational activities have been used to foster students’ emotional understanding, interpersonal communication, and social awareness (Briones et al., 2022; Wang et al., 2025). Compared with courses centered primarily on instruction and conceptual learning, drama-based social-emotional learning asks students to engage with others’ situations through bodily action, shifts in role, and collaborative creation. It has therefore been regarded as a more experiential approach to the development of social-emotional competencies (Huang et al., 2023; Levett-Jones et al., 2024). The introduction of a particular course format, however, does not in itself ensure that meaningful learning will occur. The same embodied activity may generate engagement, trust, and emotional understanding, but it may also produce embarrassment, withdrawal, pressure associated with role performance, or emotional strain. The more important question is therefore not whether drama activities have educational value, but under what relational and pedagogical conditions students are willing to enter into them, and how bodily experiences become learning experiences that can be understood, integrated, and carried forward.

Research on drama education and social-emotional learning has established several important points. Role-play, improvisation, and collaborative creation can support emotional expression, perspective-taking, self-awareness, and interpersonal understanding. Teacher-provided autonomy support, emotional care, and task guidance have also been closely associated with students’ motivation, engagement, and satisfaction of basic psychological needs (Arda Tuncdemir, 2025; Malinina, 2024; Patzak and Zhang, 2025). Peer interaction can provide emotional support, responsiveness, and a sense of group belonging, making it an important social condition for collaborative and experiential learning (Payne et al., 2025; Verbree et al., 2025). Research on embodied cognition further suggests that emotion and meaning do not arise solely from abstract thought; bodily posture, movement, spatial position, and sensory states also participate in cognitive processes (Lyu and Deng, 2024; Winkielman et al., 2023). Taken together, these bodies of research indicate that teachers, peers, and embodied activity may each contribute to students’ social-emotional learning. They have not, however, fully explained how these elements become connected within the unfolding activity of the classroom. Three related gaps remain in current explanations: (1) Teacher support and peer support are often combined under the broad notion of a “supportive environment.” As a result, the functions performed by different actors during classroom activities, and the ways in which these forms of support relate to one another, have not been sufficiently differentiated. Although previous studies have established associations between support and positive learning outcomes, they have paid less attention to how teacher responsiveness, task structure, and peer interaction enter into students’ experiences of safety, autonomy, and belonging (Prananto et al., 2025; van Herpen et al., 2025); (2) Embodied participation is frequently treated as a condition that facilitates social-emotional learning, yet bodily activity does not necessarily produce understanding in its own right. Tension, excitement, or discomfort experienced within a role may need to be responded to, articulated, and reflected upon before it can contribute to a fuller understanding of oneself or others. There remains limited process-based explanation of when bodily experience becomes part of meaning-making, when it remains a temporary stimulus, and when it develops into an emotional burden (Hegna and Ørbæk, 2024; Madsgaard et al., 2022); and (3) Much of the existing evidence has been derived from questionnaires, pre- and post-course measures, programme evaluations, or retrospective accounts from a single group of participants. Studies have less often examined classroom interaction, student reflection, teacher interpretation, and course-design materials together (Carr et al., 2021; Slater et al., 2025). Reliance on a single source of data makes it difficult to identify possible differences between teachers’ intentions and students’ experiences. It may also obscure situations in which teacher support is insufficient, peer responsiveness is limited, or activities are poorly matched to participants. Moreover, there is limited multisite evidence concerning whether, and in what ways, different course traditions or pedagogical arrangements shape these processes.

To address these issues, this study adopted a multisite interpretive qualitative design and examined drama-based social-emotional learning courses at four universities in eastern China. Drawing on interviews with students and teaching staff, student reflective journals, classroom observations, and course documents, the study investigated how teacher support, peer interaction, and embodied participation were enacted in classroom practice and how they became interconnected. It addressed the following research questions:

RQ1: How do students experience teacher support and peer interaction in drama-based social-emotional learning courses, and how are these experiences situated within classroom practice?

RQ2: How are teacher support, peer interaction, and embodied participation connected with students’ experiences of autonomy, competence, relatedness, and social-emotional learning?

RQ3: What shared patterns, contextual variations, and limiting conditions characterize these processes across different course settings?

## 2 Theoretical review and initial analytical framework

### 2.1 Core concepts and levels of analysis

Drama-based social-emotional learning refers to a curricular process that uses role-play, improvisational creation, situational simulation, bodily expression, and collaborative reflection as its main media, enabling learners to understand themselves, comprehend others, and adjust their relational behavior through action, emotion, and interpersonal interaction (Arda Tuncdemir, 2025; Briones et al., 2022). It differs from courses centered primarily on the instruction of social-emotional knowledge and should not be equated with general drama-skills training. The former mainly emphasizes conceptual understanding, whereas the latter generally focuses on performance skills and artistic outcomes. Drama-based social-emotional learning instead examines how bodily participation and shared activity contribute to emotional understanding, perspective-taking, self-awareness, and relational negotiation. Teacher support refers to the process through which teachers create conditions for students’ participation by structuring tasks, responding to students, attending to their emotional experiences, and deciding when and how to intervene. It includes explaining tasks, demonstrating activities, and providing scaffolding, as well as responding non-judgementally to hesitation, failure, and emotional reactions (Ahmadi et al., 2023; Patzak and Zhang, 2025). Peer support refers to the relational experiences formed through attention, responsiveness, collaboration, and feedback during shared activities, rather than merely the quality of friendships or individuals’ general evaluations of the classroom climate (Payne et al., 2025; Verbree et al., 2025). Analytically, teacher support concerned instructor-led structuring and responsiveness, whereas peer support concerned reciprocal attention, continuation, and collaboration among students. Embodied participation refers to the process through which bodily movement, posture, sensation, spatial position, and rhythms of action become involved in the formation of emotion and cognition. It is distinct from the behavioral fact of having participated in a physical activity and focuses more specifically on how bodily experiences are noticed, interpreted, and connected with one’s own situation or that of others (Hegna and Ørbæk, 2024; Winkielman et al., 2023). Embodied participation refers to bodily experience itself, whereas embodied meaning-making refers to how that experience is subsequently noticed, articulated, and interpreted.

In this study, social-emotional learning is not treated as objectively measured growth in competence. It refers instead to experiences of emotional understanding, self-awareness, understanding of others, and relational coping that emerge during the course. The unit of analysis is therefore neither the individual traits of students nor the macro-level institutional system of the university, but the relational and activity processes of the classroom. The sustainability of the learning ecology refers to the processual condition through which classroom participation is maintained, adjusted, or restored across successive course activities. It does not imply stable competencies or lifelong effects verified through longitudinal follow-up.

### 2.2 Self-determination theory: how social conditions enter learners’ experiences

Self-determination theory proposes that autonomy, competence, and relatedness are basic psychological needs that support high-quality motivation and psychological development. Autonomy refers to the experience that one’s actions involve choice and personal endorsement. Competence refers to the experience of being able to deal effectively with a task. Relatedness refers to the experience of being accepted by others and maintaining meaningful connections with a group (Holmquist et al., 2024; Howard et al., 2021; Wang et al., 2024). Educational environments do not directly determine learning outcomes. Rather, they influence how students understand tasks, engage in activities, and sustain their motivation by supporting or frustrating these needs. This theory provides an important starting point for examining teacher support in drama classrooms. Autonomy support does not mean removing requirements. It involves explaining the meaning of a task, acknowledging students’ feelings, and preserving appropriate opportunities for choice. Structure support provides students with a direction for action through clear goals, comprehensible rules, and informational feedback (Ahmadi et al., 2023; Reeve and Cheon, 2021). In drama activities, students require both space for expression and a clear understanding of roles, tasks, and interactional boundaries. Excessive restriction of choice may create a sense of control, whereas insufficient structure may increase the uncertainty associated with open-ended tasks.

The theory also helps explain peer relationships. Whether students feel that their expression is acknowledged, whether they are able to contribute to a shared task, and whether they have a place within the group may involve experiences of both relatedness and competence (Shin and Johnson, 2021; Worley et al., 2023). From this perspective, teacher support and peer support are both social conditions within the classroom, but they may relate to basic psychological needs in different ways. They should not therefore be combined in advance as a single form of “positive environment.” Self-determination theory, however, commonly describes the social environment in terms of need-supportive or need-thwarting conditions and offers less explanation of how bodily movement and sensory states participate in need-related experiences and social understanding. It can explain why supportive conditions may increase students’ willingness to participate actively, but it is less able to explain how role-related posture, bodily tension, changes in movement, and spatial relationships become sources for understanding oneself and others. Embodied cognition therefore provides a complementary perspective for examining how bodily experience contributes to the formation of social-emotional meaning.

### 2.3 Embodied cognition: how bodily experience forms social-emotional meaning

Embodied cognition challenges the view of cognition as abstract processing detached from the body. It emphasizes that sensorimotor systems, bodily states, and action within the environment jointly participate in the formation of concepts, emotions, and meaning (Borghi et al., 2022; Lebert and Vilarroya, 2024; Winkielman et al., 2023). Emotional understanding depends not only on verbal labels but also on bodily cues such as breathing, muscular tension, posture, action tendencies, and spatial distance. The body is not merely a passive expression of psychological states but forms part of the cognitive process. Drama activities make the relationship between the body and meaning particularly observable in classroom settings. Learners enter roles through posture and movement, approach or avoid others within a shared space, and respond to the actions of their partners. Role-play and improvisation can provide experiential media for perspective-taking, emotional expression, and understanding the situations of others (Basit et al., 2023; Malinina, 2024; Näykki et al., 2024).

Bodily arousal, however, is not equivalent to social-emotional learning. Tension, excitement, or discomfort may remain temporary experiences when they are not noticed, expressed, and reflected upon. High-intensity simulations may also be accompanied by stress and anxiety (Ko and Kim, 2022; Madsgaard et al., 2022). Embodied cognition explains why the body participates in meaning formation, but it offers less explanation of the relational and pedagogical conditions that support the expression and organization of experience. Embodied processes therefore need to be examined within the context created by teacher arrangements, peer interaction, and classroom reflection.

### 2.4 From individual needs to the classroom learning ecology

A learning ecology perspective understands learning as a process jointly sustained by participants, relationships, activities, resources, and rules, rather than as the effect of a single instructional factor on an individual (Networked Learning Editorial Collective, 2021; Peters et al., 2022). From this perspective, teacher support, peer interaction, bodily activity, and reflective arrangements are not independent variables. They are interconnected components of classroom processes. Learning ecology analysis examines how these elements become linked within specific activities and how they influence participants’ interpretations of their course experiences. This perspective can supplement self-determination theory by addressing classroom-system relationships that the theory examines less directly. The term “ecology,” however, should not be used as a broad integration of all positive factors. Without analysis of concrete actions and relationships, learning ecology can become a descriptive label that fails to explain how an environment is organized and sustained in practice. In this study, the classroom learning ecology is therefore defined as the actual connections among course structure, teacher support, peer relationships, bodily activities, and reflective arrangements. It is not assumed to be stable, coordinated, or necessarily beneficial.

The integration of these perspectives produces a progressively layered account. Self-determination theory explains how social conditions relate to experiences of autonomy, competence, and relatedness. Embodied cognition explains how the body participates in emotional and social understanding. The learning ecology perspective places individual psychological and bodily experiences within the relationships and activity structures of the classroom. None of these perspectives can independently explain the full process. A focus only on psychological needs may overlook the body and the forms taken by interaction. An emphasis only on embodied experience may underestimate relational conditions and pedagogical organization. The use of ecological language alone may make it difficult to identify how external conditions enter students’ concrete experiences.

### 2.5 Initial sensitizing framework and research focus

Drawing on these theoretical perspectives, the study developed the initial sensitizing framework presented in Figure 1. The framework identifies four areas requiring analytical attention: (1) Concerns course and relational conditions, including teachers’ organization of tasks, their modes of response, and peer interaction; (2) Concerns experiences of basic psychological needs, including autonomy, competence, and relatedness; (3) Concerns embodied meaning-making, including bodily sensations, role experiences, emotional recognition, and reflection; and (4) Concerns participation and social-emotional learning experiences across successive course activities. These areas may be interconnected, but the framework does not assume a fixed sequence. Nor does it prescribe in advance that teacher support, peer relationships, and embodied participation will necessarily produce positive outcomes. Different conditions may support meaning-making, but they may also generate difficulties in participation, relational tensions, or emotional burdens. The specific themes, the relationships among them, and their limiting conditions were developed through data analysis rather than treated as a predetermined theoretical model to be tested.

**FIGURE 1:**
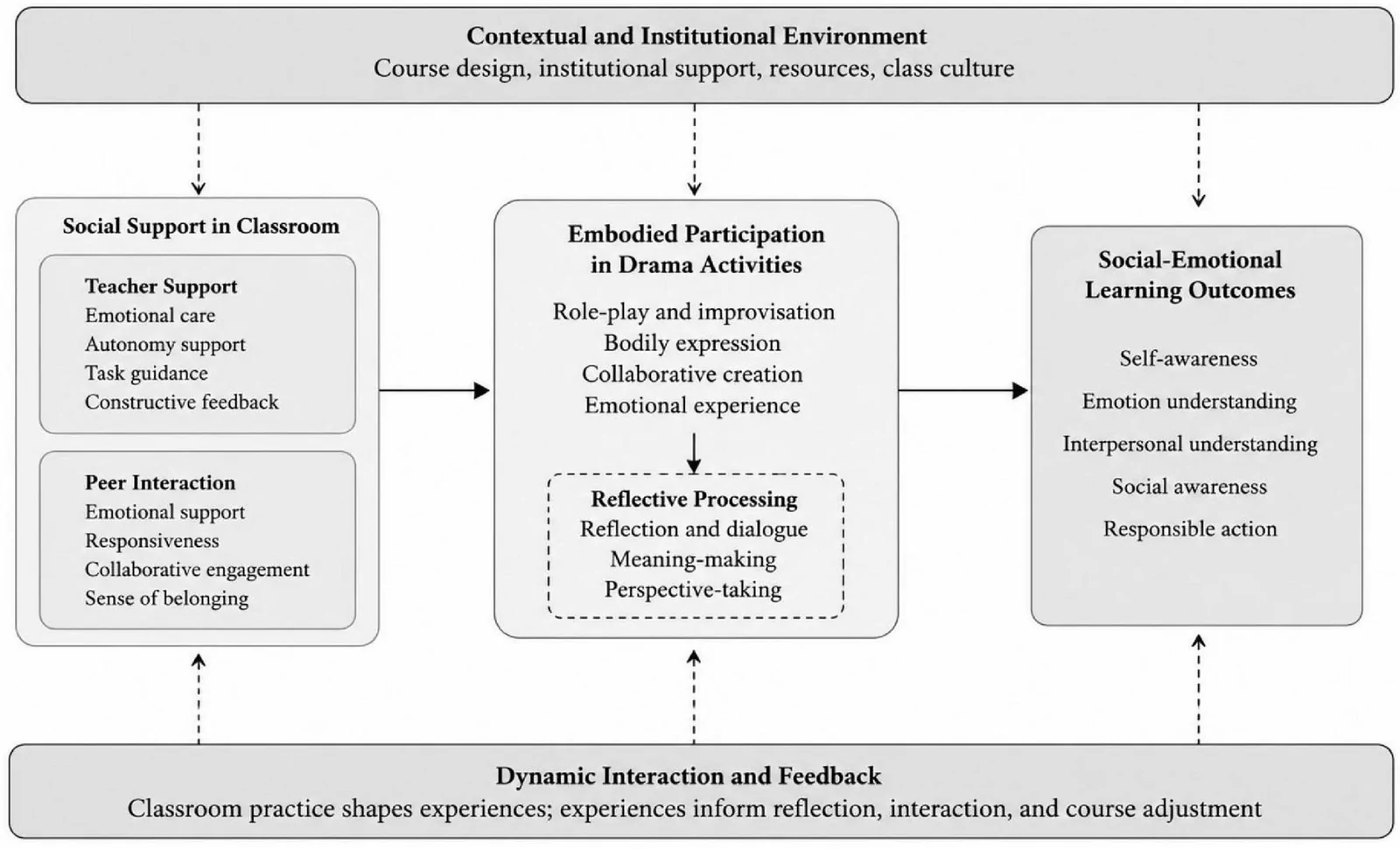
Initial sensitizing framework for drama-based social-emotional learning.

Existing research has separately established the importance of social support, basic psychological needs, and bodily participation. It has not yet adequately explained the different roles performed by teachers and peers in classroom activities, how external support enters students’ experiences through bodily and relational processes, or the course conditions under which these processes are sustained or constrained (Castro-Alonso et al., 2024; Howard et al., 2021; Peters et al., 2022). Bringing accounts from multiple participant groups, classroom observations, reflective journals, and course documents into the same analysis allows the focus to move beyond correlations among factors towards concrete interaction and the formation of meaning.

## 3 Methods

### 3.1 Epistemological position and research design

This study was grounded in an interpretivist epistemology and a constructivist view of knowledge. Students’ and teachers’ understandings of teacher support, peer interaction, embodied participation, and social-emotional learning were treated as contextualized meanings formed through course activities, interpersonal relationships, and retrospective accounts, rather than as fixed facts existing independently of their settings. The study focused on how participants made sense of these experiences and why similar experiences took different forms across course contexts. A multisite interpretive qualitative design was adopted, with data collected from drama-based social-emotional learning courses at four comprehensive universities in eastern China. The four universities were treated as research settings with different course traditions, rather than as four separate cases from which independent theoretical models would be developed. The analysis primarily examined patterns of meaning across the settings. School codes were also used to explore how themes were expressed and under what conditions they held across different course environments.

The data were analysed using reflexive thematic analysis. This approach was suitable for integrating interviews, reflective journals, observation records, and course documents and for developing themes organized around central patterns of meaning across the full dataset (Braun and Clarke, 2006; Braun and Clarke, 2019; Braun and Clarke, 2021). Self-determination theory and embodied cognition provided sources of theoretical sensitivity, directing attention towards basic psychological needs, bodily experience, and social-emotional meaning without constituting a predetermined coding framework. Materials that did not fit the dominant interpretation, together with experiences of participation difficulty and constraint, were also retained in the analysis.

### 3.2 Research settings and participants

The four universities were assigned the codes A, B, C, and D. Each had offered drama-based social-emotional learning, educational drama, or related experiential courses for at least three consecutive semesters. The courses were open to undergraduate students from different years and disciplinary backgrounds. Each course ran for 16 weeks, with two class periods per week, and included role-play, improvisational creation, mirroring exercises, situational simulations, group games, and collective reflection. Although the four courses shared an experiential basis, they differed in emphasis. The courses at the four universities placed greater emphasis, respectively, on improvisational expression, structured role work, reflective integration, and group activities. These differences created distinct research settings. Purposive maximum-variation sampling was used (Patton, 2015). Sufficient experience of classroom participation was the basic inclusion criterion. Students who had withdrawn or been absent for extended periods were therefore not included. Variation was also sought in university, participant role, gender, year of study, disciplinary background, previous drama experience, and level of course engagement. The final sample consisted of 24 students and eight members of the teaching staff. Six students, one lead instructor, and one teaching assistant or instructional support staff member were selected from each university. Students were assigned the codes SA01–SD06, and teaching staff were coded T01–T08. Detailed participant characteristics are presented in Table 1 and Supplementary Table 1.

**TABLE 1:** Course characteristics across the four research settings.

Research setting	Primary course formats	Instructional organization	Distinctive feature
University A	Improvisation and embodied expression	Greater emphasis on open-ended tasks and movement-based exploration	Relatively high degree of curricular openness
University B	Structured role-play and scenario-based scripts	Clearly specified role expectations and situational boundaries	Relatively high degree of instructional structure
University C	Psychodrama and collective reflection	Greater emphasis on post-activity discussion and reflective processing	Emphasis on the interpretation and integration of experience
University D	Group games and embodied warm-up activities	Greater emphasis on warm-up activities and collective participation	Emphasis on shared activity and group involvement

The first researcher was responsible for the study design, communication with the four universities, and the main analysis. The researcher was familiar with the course contexts but was not involved in teaching the courses or assessing students’ academic performance. Recruitment and interviews were conducted by research assistants who had no teaching role in the courses. The second researcher acted as a critical partner and reviewed selected raw materials, codes, thematic structures, and cross-setting comparison records. Through reflexive memos and three peer discussions, the research team continually examined whether prior assumptions about the positive educational value of drama were shaping the interpretation of the data.

### 3.3 Data collection

Data were collected across a complete 16-week course cycle during the autumn semester of 2025. The dataset included semi-structured interviews, student reflective journals, non-participant classroom observations, and course-related teaching documents. A total of 32 individual interviews were completed. The 24 student interviews lasted approximately 40 min on average, while the eight teaching staff interviews lasted approximately 50 min. The total interview time was approximately 22.7 h. With participants’ consent, all interviews were audio-recorded and transcribed verbatim, producing approximately 190,000 Chinese characters of textual data. The student interviews focused on teacher guidance, peer interaction, bodily and emotional experiences, basic psychological needs, social-emotional learning, and experiences of difficulty. Interviews with teaching staff addressed course design, classroom organization, student responses, and implementation constraints. The interview guides are provided in Supplementary Table 2. Each of the 24 students submitted one reflective journal of approximately 1,000–2,000 Chinese characters. The journals supplemented aspects of bodily sensation, emotional change, collaborative experience, and related experiences beyond the course that participants found difficult to express fully during interviews. Twenty-four classroom observations were conducted, with six observations at each university. The observations were distributed across the early, middle, and later stages of the course. Each observation lasted approximately 90 min, producing a total of approximately 36 observation hours. Attention was given to teacher responses, bodily activities, spatial relationships, peer collaboration, classroom participation, and reflection following the activities. Sixteen course documents were also collected, with four documents from each university. These included course syllabi, teaching plans, activity designs, and reflective materials.

The different data sources were not expected to converge completely. Consistent materials strengthened the interpretive basis of the analysis. Discrepancies were used to examine possible differences among course design, teachers’ understandings, students’ experiences, and classroom implementation. All participants included in the analysis were adults and provided written informed consent. The study received ethical review and approval from the researchers’ institution and was conducted in accordance with the Declaration of Helsinki and relevant ethical standards for research involving human participants. Codes were used for all universities and participants, and identifying information was removed before analysis. Audio recordings, transcripts, and reflective journals were accessible only to authorized members of the research team. Classroom observation records did not identify or directly quote individuals who had not participated in the interviews.

### 3.4 Data analysis

NVivo 14 was used for data management, code retrieval, and the documentation of analytical decisions. NVivo was used for organization and retrieval rather than frequency-based textual analysis. Quotations were selected for their relevance to thematic meaning, variation, or constraining cases rather than their frequency. The analysis followed the six-phase procedure proposed by Braun and Clarke (2006); Braun and Clarke (2019); Braun and Clarke (2021). Movement between phases was iterative rather than mechanically sequential. (1) The researchers repeatedly read the interview transcripts, reflective journals, and observation notes, recording preliminary patterns of meaning, contradictory materials, and their own interpretive tendencies; (2) The data were coded segment by segment, producing 187 initial codes. The same segment could be assigned to more than one code. Coding addressed instructional organization, interactional responses, bodily and emotional experiences, participation difficulties, differences in perspective, and constraining experiences; (3) Related codes were organized into candidate themes. Attention was given to whether the codes collectively explained a pattern of experience organized around a central meaning, rather than merely reproducing the topics covered in the interviews; (4) Candidate themes were repeatedly compared with the complete dataset. The researchers examined coherence within themes, distinctions between themes, perspectives expressed by different participants, and the inclusion of contradictory or negative cases; (5) The central analytical claim, scope, and relationships of each theme were defined; and (6) School codes were used to compare how the same themes were expressed across the four course settings, after which the final analytical narrative was developed. The complete analytical procedures and examples showing the relationships among data extracts, codes, and themes are provided in Supplementary Table 3.

### 3.5 Research rigor

Research quality was addressed through credibility, transferability, dependability, and confirmability (Lincoln and Guba, 1985). (1) Credibility was supported through the inclusion of multiple participant groups, comparison across data sources, negative-case analysis, and participant feedback. The researchers returned summaries of the themes to four students and two members of the teaching staff. Participants were invited to identify factual errors, important omissions, or misunderstandings of their experiences rather than to confirm a single authoritative interpretation; (2) Transferability was supported by providing contextual information about the courses, participant characteristics, activity processes, and differences among the research settings; (3) Dependability was supported through a complete audit trail, including the original data, versions of the coding structure, records of theme development, analytical memos, and a cross-setting comparison matrix; and (4) Confirmability was addressed through reflexive journals, review by the critical partner, and the examination of competing interpretations. These procedures were used to consider how the researchers’ identities, theoretical positions, and prior assumptions influenced the interpretive process.

## 4 Findings

Reflexive thematic analysis of the interviews, student reflective journals, classroom observations, and course documents generated three interrelated overarching themes and six subthemes: participatory safety and bounded autonomy through teacher scaffolding; peer connection and group belonging through embodied collaboration; and emotional resonance and self-awareness through embodied meaning-making. All three themes were present across the four course settings, although their forms varied according to activity structure, teachers’ modes of intervention, and the state of group interaction. The thematic structure is presented in Table 2, and representative evidence from multiple data sources is provided in Supplementary Table 4.

**TABLE 2:** Core themes, subthemes, and central meanings.

Core theme	Subtheme	Central meaning
Participation safety and bounded autonomy through teacher scaffolding	Non-evaluative guidance and participation safety	Teachers’ responses reduced students’ concerns about public failure and negative evaluation.
	Structural scaffolding and bounded autonomy	Clear activity boundaries and opportunities for expressive choice jointly supported students’ entry into the activities.
Peer connection and group belonging through embodied collaboration	Nonverbal collaboration and the development of mutual trust	Gaze, movement continuation, and rhythmic coordination enabled trust to develop through shared activity.
	Peer acceptance and group belonging	When individual expression was acknowledged and extended by others, connection expanded from dyads to small groups or the wider class.
Emotional resonance and self-awareness through embodied meaning-making	Embodied empathy and emotional resonance	Bodily responses experienced in role enabled students to develop a felt understanding of others’ situations.
	Bodily awareness, transfer, and risk	Bodily signals supported self-understanding and the application of classroom perspectives in other situations, while also carrying the possibility of discomfort or emotional overload.

### 4.1 Teacher scaffolding for safety and autonomy

#### 4.1.1 Non-evaluative guidance and participatory safety

Students across all four settings reported that teachers rarely judged a performance directly as “right” or “wrong.” Instead, they used follow-up questions to encourage further exploration. SB02 explained: The teacher never said, “You performed it incorrectly.” It was always, “How else could you perform it?” or “What would you do if you were this person?” Questions like these made me willing to try. Open-ended questions changed how students assessed the risks of classroom participation. Expression was no longer understood as a public performance that had to succeed at the first attempt, but as something that could be revised and explored further. Classroom observations showed that lead instructors at all four universities used prompts such as “What other possibilities are there?” and “What do you think this character is feeling at this moment?” As the courses progressed, direct teacher intervention became less frequent, while students increasingly discussed roles, allocated tasks, and adjusted their forms of expression themselves.

Individualized attention also contributed to participatory safety. Some students interpreted teachers’ enquiries about their immediate condition as concern for them as individuals rather than merely for task completion. This attention allowed students who were temporarily hesitant or not ready to participate fully to retain a place within the activity rather than having to withdraw immediately.

#### 4.1.2 Structure and bounded autonomy

Non-evaluative responses created a stable space for participation only when combined with a comprehensible activity framework. SA04 stated: The teacher would give us a very clear situation, but we decided how to persuade someone, what approach to use, and what tone to take. That kind of freedom within boundaries actually made me feel more secure. The autonomy evident in the data was not unrestricted freedom. Students experienced choice in how they expressed themselves after the roles, tasks, and time boundaries had been clarified. The more explicit role structures used at University B helped students understand what they were expected to accomplish. The improvisational activities at University A left more room for spontaneous action among students who had already become familiar with the classroom rules. Observation records reflected this contrast, with clearer role and task boundaries at University B and greater open-ended action at University A.

This combination did not appear uniformly across the dataset. Sometimes, “Act however you like” is more difficult than being told what you are supposed to portray, because I do not know where the boundaries are(SA06). For students with limited drama experience or those who had not yet become comfortable with bodily expression, excessive openness increased uncertainty. Overly detailed instructions, however, could turn participation into mechanical task completion. Teacher scaffolding therefore involved continuous adjustment rather than a fixed increase or reduction in guidance. Participatory safety provided an initial basis for entering shared activities, but the development of subsequent relationships depended on how peers responded to one another.

### 4.2 Peer connection and belonging

#### 4.2.1 Non-verbal coordination and trust

When describing a mirroring activity, SC05 explained that you have to look at the other person very carefully and imitate every movement she makes. That kind of focused attention is not just a casual glance. You are genuinely “seeing” the other person. Focused attention allowed students to feel noticed and enabled partners to begin recognizing one another’s rhythms of movement. SD06 understood trust in paired improvisation as the partner’s willingness and ability to continue what the other person had initiated: At first, I was afraid that if I made a mistake, my partner would not know how to respond. Later, I realized that whatever I did, my partner could carry it forward. It felt like, “Whatever you do is fine. I will follow you.” The continuous development of one another’s movements created relational safety and allowed students to recognize that their own expression could contribute to the shared task.

Classroom observations showed that students’ bodily orientation was directed mainly towards the teacher during the early stages of the courses. From the middle stages onwards, postures facing peers, nodding, leaning forward, and gestural responses became more frequent. Group collaboration also became less dependent on immediate teacher instructions.

#### 4.2.2 Peer acceptance and belonging

The trust formed through repeated collaboration gradually extended from coordinated movement to verbal expression and discussion. Students became more willing to raise different views and better able to tolerate uncertainty during role activities. Describing a group activity at University D, as SD02 stated: We warmed up together, laughed together, and did movements that looked rather silly. It was precisely those moments of being “silly” together that made me feel that I was part of the class. The scope of belonging differed across settings. Students at Universities A and B more often described coordination and mutual understanding with a partner or small group. Collective reflection at University C and group games at University D more readily extended connection to the class as a whole. Differences among the settings are presented in Table 3 and Supplementary Table 5.

**TABLE 3:** Manifestations of the three themes across the four course settings.

Analytical dimension	University A	University B	University C	University D
Teacher scaffolding	Open-ended guidance during improvisational tasks	Clearly defined role boundaries and task structures	Reflective questioning following embodied activities	Low-pressure support during group activities
Peer relationships	Dyadic coordination developed through improvisation and mirroring	Small-group collaboration developed through role-based tasks	Emotional trust developed through psychodrama and collective reflection	Group belonging emerged relatively early through collective games
Embodied experience	Greater prominence of spontaneous bodily responses	Greater prominence of situational and role immersion	Greater emphasis on emotional processing and meaning integration	Greater intensity of bodily activity and collective participation
Principal limitations	Open-ended tasks could create uncertainty about activity boundaries	Highly structured tasks could restrict spontaneous expression	Emotional intensity and time for reflection could become imbalanced	Activities could remain at the level of positive atmosphere without deeper reflective processing

Shared participation did not automatically produce peer support. Activities could intensify embarrassment when peers focused mainly on performance quality, failed to respond to another person’s movements, or entered the activity with existing relational tensions. Peer support depended on mutual waiting, attention, and the continuation of one another’s contributions, rather than simply placing students in the same group. Relational safety further shaped whether students were willing to enter more demanding bodily and emotional experiences.

### 4.3 Embodied meaning-making

#### 4.3.1 Embodied empathy and emotional resonance

SA03 described the bodily state experienced while playing the role of an excluded person: My whole body became stiff. My shoulders were tense, I did not know where to put my hands, and I could not look anyone in the eye. It was not something I had “thought up.” My body reacted on its own. Posture, spatial position, and tone of voice within the role made another person’s situation more than a story to be understood. It became a bodily state that students experienced temporarily.

Discussion after the activities allowed students to recognize that feelings of tension, powerlessness, or isolation could be understood and responded to by others. The structured roles at University B strengthened students’ immersion in the situation. University A placed greater emphasis on immediate reactions during movement, while University C relied more heavily on post-activity reflection to help students organize the emotional meaning of their experiences.

#### 4.3.2 Bodily awareness, transfer, and risk

Embodied experiences also led students to pay closer attention to their bodily signals and habitual ways of interacting. SC03 described a change that occurred during a disagreement with a roommate:I now stop and think about what her body is showing at that moment—whether she is tense or relaxed. This perspective changed me from thinking, “I need to argue back,” to thinking, “I want to understand what state she is in.” This change involved students drawing on classroom experiences in new situations. It did not indicate that they had developed a stable ability to resolve all relational problems. Several reflective journals recorded that students had begun to notice bodily signals such as biting their lips, stiffening their shoulders, or avoiding eye contact, and to connect these signals with tension, resistance, or unease.

Bodily participation could also exceed what students felt able to manage. The emotion was too intense. It felt as though the character had swallowed me. It took me several minutes to come out of that state (SC06). T06 similarly noted that high-intensity emotional activities required careful control of depth, together with sufficient time for students to step out of role and settle emotionally. Without these conditions, bodily experience could remain a source of discomfort or burden rather than entering a subsequent process of meaning-making. Embodied learning therefore contained both the possibility of generating understanding and the risk of emotional dysregulation. The three themes were present across all four course settings, but their main expressions and constraining experiences were not identical. The principal comparisons among the four settings are presented in Table 3.

### 4.4 Cross-theme integration: interconnected participation

Comparison across themes showed that the three themes did not operate independently but were interconnected across course activities. Teachers’ non-evaluative responses and appropriately structured tasks were associated with lower concerns about participation. Once students entered shared embodied activities, they gradually developed peer connection through sustained attention, the continuation of one another’s movements, and collaborative action. When students’ expressions were taken up and responded to by peers, they became more willing to enter a role and express emotion. Bodily responses that were subsequently discussed and reflected upon could be interpreted as insights into themselves and others. Some participants linked experiences in one activity with their willingness to participate in later activities. SB04 summarized this change: During the first activity, I was simply completing the task. Once I realized that my expression was accepted and that my peers were genuinely listening to me, I was more willing to engage the next time.

This process did not develop in a single direction, nor did it occur consistently across all students and activities. Unclear boundaries, failures in peer responsiveness, role-related pressure, and emotional overload were associated with reduced participation in subsequent activities. Differences among the course settings mainly concerned how each part of the process was enacted and did not provide a basis for ranking the effectiveness of the courses. The qualitative associations among the themes are summarized in Figure 2. Data sources and constraining evidence for each part of the process are presented in Supplementary Table 6.

**FIGURE 2:**
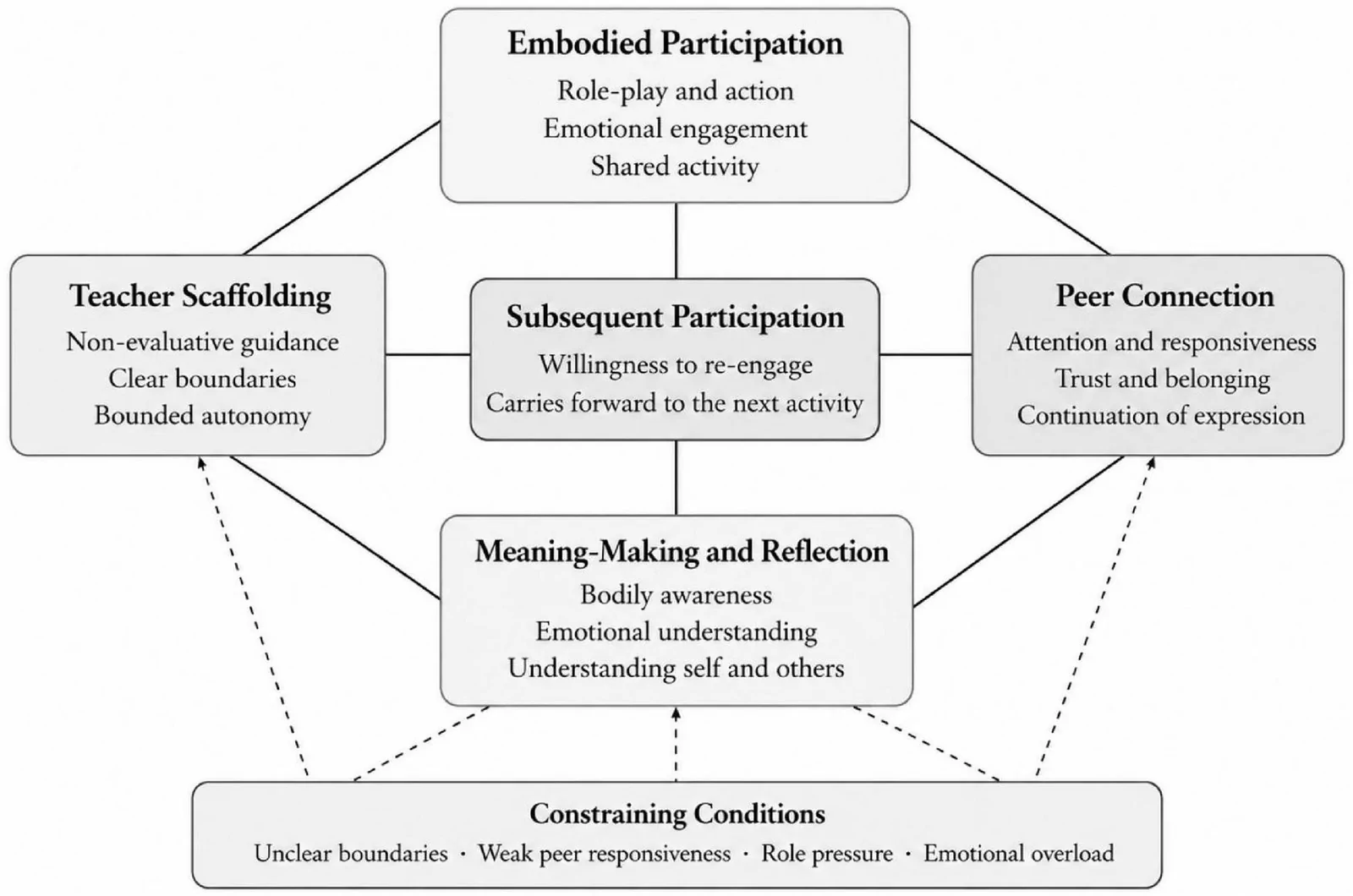
Interconnected participation in drama-based social-emotional learning.

## 5 Discussion

### 5.1 Teacher scaffolding: safety and bounded autonomy

Teacher support served two interdependent functions in drama-based social-emotional learning. First, open-ended questioning, non-evaluative responses, and attention to students’ immediate conditions reduced the evaluative pressure associated with public expression. Second, roles, tasks, and activity rules provided comprehensible boundaries for participation. The first function made students more willing to try, while the second gave them a clear way to enter the activity. The absence of either could weaken their willingness to participate. This finding is consistent with research in Chinese higher education showing a positive relationship between autonomy support and students’ agentic engagement (Gan et al., 2026). Previous analyses have also suggested that autonomy support and structure are not mutually exclusive. Structure helps students understand the task, while autonomy support allows them to retain choice and personal endorsement within it (Patzak and Zhang, 2025).

The present study extends this understanding to drama classrooms characterized by public expression, bodily participation, and uncertainty within roles. In such settings, autonomy does not require the teacher to withdraw. Insufficient guidance may be experienced as unclear boundaries, while excessive prescription may restrict students’ expressive space. Teacher scaffolding is therefore better understood as a pedagogical practice adjusted in relation to the activity context. In practice, this involved both oral guidance and activity structuring: teachers clarified roles, tasks, and boundaries while leaving choices of strategy, tone, and expression to students. At the beginning of a course, clear rules and low-risk tasks can reduce the difficulty of initial participation. Once students become familiar with the activities, direct intervention can be reduced and more expressive decisions can be returned to them. Activities involving stronger emotions may require renewed support and time for emotional settling. The central issue is not the amount of support, but whether structure, choice, and the timing of intervention are appropriate to students’ experiences and the intensity of the activity.

### 5.2 Peer support and group belonging

Peer support was not always a social resource that existed before students entered the course. Mirroring, improvisation, the continuation of one another’s movements, and group games required students to observe, wait for, and respond to others. Through repeated interaction, students gradually recognized that their movements could be noticed, their expressions could be continued, and mistakes would not immediately interrupt the relationship. Peer trust could therefore develop through shared activity rather than depending only on familiarity or pre-existing friendship. Research in higher education has shown that organized peer interaction and group activities can provide concrete relational conditions for a sense of belonging (Kelly et al., 2025). Although belonging in higher education has been defined and measured in different ways, interpersonal connection and a sense of acceptance are commonly treated as central components (Dias-Broens et al., 2024). Belonging has also shown consistent associations with students’ academic and psychological outcomes (van Kessel et al., 2025). The present study adds an account of how this experience was formed through action. Sustained attention communicated recognition, waiting preserved the other person’s expressive rhythm, and the continuation of movement confirmed that an individual contribution could become part of a shared task.

Peer connection also carried experiences of both group belonging and situated competence. When a student’s movement or role expression was acknowledged by peers, the student experienced acceptance and recognized that their action could contribute to the shared task. Previous research has similarly linked positive social interaction with a sense of belonging to a professional or learning community (Polmear et al., 2024). Placing students in the same group, however, was not sufficient to generate support. Shared activities could intensify embarrassment and withdrawal when peers focused on performance quality, overlooked another person’s movements, or brought existing relational tensions into the activity. The emergence of peer support depended on whether the activity required and protected mutual attention, responsiveness, and negotiation. Mirroring and paired improvisation enabled this by requiring students to attend to and continue one another’s actions rather than simply perform alongside one another.

### 5.3 Embodied meaning-making and interconnected participation

Bodily participation provided an experiential entry into social-emotional learning that differed from conceptual instruction. Research on embodied learning suggests that movement, sensation, and interaction with the environment participate in learning rather than functioning as additions external to cognition (Jusslin et al., 2022). Related studies have also shown that taking on different roles can expose students to complex positions and relational situations (Visscher, 2024). In this study, posture, spatial position, movement rhythm, and bodily tension made another person’s situation temporarily available as a felt state. These experiences also directed students’ attention towards their own emotions and habitual ways of interacting. Embodied participation therefore provided the experiential material from which social-emotional understanding could develop, but the experience did not acquire such meaning automatically. Its significance was shaped as students’ bodily responses were noticed, taken up by others, articulated in discussion, and reconsidered through reflection. Reviews of simulation-based education have similarly identified post-activity reflection and debriefing as important links between experience and learning (Fegran et al., 2023). The four course settings placed different emphases on immediate bodily response, situational immersion, reflective organization, and shared participation. These differences suggest that the educational significance of a course form depends on whether bodily experience is given an appropriate point of entry and sufficient space for subsequent interpretation.

Teacher scaffolding, peer connection, embodied meaning-making, and participation were interconnected rather than arranged in a fixed sequence. Participants sometimes linked experiences of safety and responsiveness with their willingness to participate in later activities.. Students’ modes of participation were also associated with peer interaction and teachers’ decisions about intervention. This process could be interrupted. Breakdowns did not take a single form. For some students, unclear boundaries made it difficult to know how far they could go in a role. Others became more self-conscious when a partner failed to respond, while emotionally intense activities sometimes left students carrying the experience beyond the point at which it remained productive. A meta-analysis of simulation learning in higher education has likewise shown that activity design can affect students’ stress, anxiety, and confidence (Oliveira Silva et al., 2023). Bodily participation can generate social-emotional meaning, but it requires clear activity boundaries, opportunities to withdraw, and structured reflection.

### 5.4 Theoretical contributions and course design

This study provides three theoretical implications: (1) It distinguishes the different positions occupied by teacher support and peer support within the drama classroom. Teacher support primarily involved the regulation of activity structure, expressive risk, and emotional intensity. Peer support emerged through shared activity, the continuation of one another’s expressions, the development of trust, and the formation of group belonging. The two forms of support were interconnected, but they could not be combined into a single supportive environment or treated as substitutes for one another; (2) Basic psychological needs appeared in drama classrooms as concrete interactional experiences. Students experienced autonomy within comprehensible boundaries. When their expressions were taken up by others, they experienced both relatedness and competence. When bodily sensations were discussed and named, they developed a clearer understanding of themselves and others. This interpretation places need-related experiences within bodily action and relational interaction, showing how external support was perceived through specific classroom events; and (3) The interconnected participation pattern suggests that the sustainability of a classroom learning ecology is not a stable quality that exists in advance. It is repeatedly formed and adjusted across successive activities. Safe attempts within clear boundaries were associated with stronger participation, whereas evaluation, neglect, and emotional overload were associated with withdrawal. Students are not only recipients of support. Through responding to or overlooking others, they also participate in the formation of classroom relationships.

Course design should move from low-risk, clearly bounded activities toward greater expressive choice and interactional demands. Early activities can specify roles, rules, and time boundaries, while later activities retain these boundaries but allow greater choice of strategy, movement, and role interpretation. Activities involving stronger emotions should include opportunities to pause, withdraw, relax physically, and reflect. Assessment should not center on performance skill or the visible intensity of emotional expression. Greater attention can be given to whether students respond to peers, explain their expressive choices, recognize bodily sensations, and organize the meaning of their experiences.

These findings should also be interpreted within the Chinese higher-education context. Norms concerning teacher authority, face, and group harmony may shape the salience of non-evaluative guidance, bounded autonomy, and peer acceptance, although these cultural influences were not directly examined here (Chan and Smith, 2024). Cross-cultural SDT research nevertheless suggests that autonomy support remains relevant across Eastern and Western contexts (NalipayKing et al., 2020).

### 5.5 Limitations and future research

Whereas, this study still remains several limitations: (1) The research settings were limited to elective courses at four comprehensive universities in eastern China. Although the courses differed in pedagogical emphasis, they were situated within similar higher education and cultural contexts. Further research is needed to examine whether the present interpretation applies to arts universities, vocational institutions, compulsory courses, and other cultural settings. The shared Chinese cultural context also limits transferability to settings with different norms of authority, autonomy, and peer interaction; (2) The study did not include students who withdrew from the courses or were absent for extended periods, creating possible selection bias toward students who remained engaged. The findings may therefore underrepresent more severe forms of disengagement or disruption. Future studies could intentionally follow experiences of withdrawal, resistance, and sustained low participation and examine the organizational and support conditions surrounding them; (3) The interviews and reflective journals contained retrospective interpretations that may have been affected by memory reconstruction and social desirability. Classroom observations provided evidence of actual interactions, but only part of the course period at each university was observed. Changes after the courses ended were not followed. Cross-semester observations, repeated interviews, continuous journals, and video-based interaction analysis could be used to examine how peer connection, bodily awareness, and experiences beyond the classroom change over time; (4) The researchers’ familiarity with the four course contexts supported an understanding of the pedagogical settings but may also have strengthened expectations concerning supportive processes. Reflexive memos, negative-case analysis, and critical-partner review helped the research team examine this influence, but they could not remove the role of researcher positioning in the development of the themes; and (5) The recursive participation process is a contextual interpretation developed from multiple sources of qualitative data. It cannot establish the magnitude of the relationships among its components or establish temporal precedence or causal relationships. Future research could develop longitudinal or mixed-methods designs while retaining attention to classroom context and constraining experiences. Such studies could further examine the relationships among teacher scaffolding, peer connection, embodied meaning-making, and subsequent participation.

## 6 Conclusion

This study examined the relationships among teacher support, peer interaction, and embodied participation in drama-based social-emotional learning courses at four universities in eastern China. The analysis drew on interviews, student reflective journals, classroom observations, and course documents. Three interrelated themes were developed: participatory safety and bounded autonomy through teacher scaffolding; peer connection and group belonging through embodied collaboration; and emotional resonance and self-awareness through embodied meaning-making. The findings indicate that students’ participation across successive course activities was not determined by any single teaching method. It was closely related to teachers’ responses and activity structures, peers’ attention to and continuation of one another’s contributions, and whether bodily experiences could be expressed and reflected upon. Teachers’ non-evaluative responses and appropriate levels of structure helped reduce concerns about public expression while preserving necessary space for student choice. Peer connection gradually developed through sustained attention, waiting, the continuation of movement, and collaborative activity, allowing individual expression to become part of a shared process. Through discussion and reflection, role-based actions and bodily sensations could be interpreted as sources of understanding of the self, emotion, and the situations of others. Safety, responsiveness, and meaning developed in earlier activities were related to how students participated in later activities. This process did not progress consistently and could be constrained by unclear boundaries, insufficient peer responsiveness, or excessive emotional intensity. Drama-based social-emotional learning is therefore better understood as interconnected relational, embodied, and reflective classroom processes rather than as a course form in which bodily activity or emotional experience naturally produces positive outcomes.

## Data Availability

The original contributions presented in the study are included in the article/Supplementary material, further inquiries can be directed to the corresponding authors.
