# Supplementary material for: How teacher support, peer connection, and embodied meaning-making interact in drama-based social-emotional learning in higher education
Source: Front Psychol. 2026 Sep 29;17:1963943. doi: 10.3389/fpsyg.2026.1963943 (PMC13658177; doi:10.3389/fpsyg.2026.1963943)
Supplement: Supplementary file 1 [file Supplementary_file_1.docx]

**Supplementary Materials**

***Supplementary Table S1. Participant Characteristics***

**Table S1-A. Student Participants**

| Participant ID | Gender | Year of study | Disciplinary background | Research setting | Prior drama experience | Self-rated engagement |
| --- | --- | --- | --- | --- | --- | --- |
| SA01 | Female | Year 1 | Humanities and social sciences | A | No | Moderate |
| SA02 | Male | Year 1 | Natural sciences | A | No | Low |
| SA03 | Female | Year 2 | Humanities and social sciences | A | Yes | High |
| SA04 | Male | Year 2 | Natural sciences | A | No | Moderate |
| SA05 | Female | Year 3 | Humanities and social sciences | A | Yes | High |
| SA06 | Male | Year 3 | Natural sciences | A | No | Low |
| SB01 | Female | Year 1 | Humanities and social sciences | B | No | Moderate |
| SB02 | Male | Year 1 | Natural sciences | B | No | High |
| SB03 | Female | Year 2 | Humanities and social sciences | B | Yes | High |
| SB04 | Male | Year 2 | Natural sciences | B | No | Moderate |
| SB05 | Female | Year 3 | Humanities and social sciences | B | No | Low |
| SB06 | Male | Year 3 | Natural sciences | B | Yes | Moderate |
| SC01 | Female | Year 1 | Humanities and social sciences | C | No | Low |
| SC02 | Male | Year 1 | Natural sciences | C | No | Moderate |
| SC03 | Female | Year 2 | Humanities and social sciences | C | Yes | High |
| SC04 | Male | Year 2 | Natural sciences | C | No | Moderate |
| SC05 | Female | Year 3 | Humanities and social sciences | C | No | High |
| SC06 | Male | Year 3 | Natural sciences | C | Yes | Moderate |
| SD01 | Female | Year 1 | Humanities and social sciences | D | No | High |
| SD02 | Male | Year 1 | Natural sciences | D | No | Moderate |
| SD03 | Female | Year 2 | Humanities and social sciences | D | Yes | Moderate |
| SD04 | Male | Year 2 | Natural sciences | D | No | Low |
| SD05 | Female | Year 3 | Humanities and social sciences | D | No | High |
| SD06 | Male | Year 3 | Natural sciences | D | Yes | Moderate |

***Note:*** *Engagement levels were based on students self-reports and were used solely to increase the diversity of participant experiences.*

**Table S1-B. Teaching Staff Participants**

| **Participant ID** | **Gender** | **Research setting** | **Years of teaching experience** | **Role** | **Primary responsibilities** |
| --- | --- | --- | --- | --- | --- |
| T01 | Female | A | 12 | Lead instructor | Course design and facilitation of improvisational and embodied activities |
| T02 | Male | A | 5 | Teaching assistant | Small-group facilitation and individualized participation support |
| T03 | Male | B | 8 | Lead instructor | Role specification and scenario development |
| T04 | Female | B | 3 | Teaching assistant | Role coordination and support for within-group relationships |
| T05 | Female | C | 15 | Lead instructor | Facilitation of psychodrama and collective reflection |
| T06 | Male | C | 6 | Instructional support staff | Preparation of reflective materials and support for emotional safety |
| T07 | Female | D | 10 | Lead instructor | Facilitation of group games and development of the classroom climate |
| T08 | Male | D | 4 | Teaching assistant | Facilitation of warm-up activities and small-group interaction |

***Supplementary Table S2. Semi-Structured Interview Guides***

**Table S2-A. Student Interview Guide**

| **Module** | **Main questions** |
| --- | --- |
| Overall course experience | Reasons for enrolling in the course, expectations before participation, and overall experiences of the course |
| Teacher support | How instructors facilitated the activities; responses that were experienced as helpful or difficult |
| Peer interaction | Experiences of collaboration, peer responses, and positive or challenging interactions |
| Embodied participation | Bodily and emotional experiences during role-play, improvisation, and mirroring activities |
| Basic psychological needs | Opportunities for choice, experiences of competence, and sense of belonging in the classroom |
| Social-emotional learning | Understandings of self, others, and relationship management; connections between course experiences and situations beyond the course |
| Limiting experiences | Difficulties in participation, resistance, regrets, and needs that were not adequately addressed |

**Table S2-B. Teaching Staff Interview Guide**

| **Module** | **Main questions** |
| --- | --- |
| Course design | Course philosophy, activity sequencing, and the intended role of drama-based approaches |
| Teacher support | Judgements regarding when to intervene and when to step back; responses to students experiencing difficulty |
| Peer interaction | Group composition, patterns of interaction, and classroom relationships |
| Embodied learning | Emotional, cognitive, and behavioral manifestations during embodied activities |
| Contextual conditions | The influence of institutional course traditions and course organization on implementation |
| Limitations and improvement | Implementation challenges, potential risks, and possible course adjustments |

***Supplementary Table S3. Analytical Procedures and Evidence Chain***

**Table S3-A. Analytical Principles**

| **Analytical stage** | **Operational principle** |
| --- | --- |
| Data familiarization | Read all data sources in full and record reflexive memos |
| Initial coding | Remain close to the meanings expressed in the data; allow a single data segment to receive multiple codes |
| Theoretical sensitization | Use theory to enhance analytical sensitivity without allowing it to predetermine codes or themes |
| Theme development | Require each theme to contain a clearly articulable central analytical claim |
| Theme review | Examine the complete dataset, divergent perspectives, negative cases, and thematic boundaries |
| Cross-setting comparison | Compare how themes were manifested across settings without ranking the four universities by effectiveness |
| Data traceability | Retain participant identifiers, data-source types, and locations in the original dataset |
| Theme reporting | Use participant numbers to indicate breadth of coverage, not to determine thematic importance by frequency |

**Table S3-B. Examples of the Data–Code–Theme Evidence Chain**

| **Original data extract** | **Initial codes** | **Aggregated meaning** | **Corresponding theme or subtheme** |
| --- | --- | --- | --- |
| The teacher never said, You performed it incorrectly. Instead, she always asked, How else could you perform it? (SB02) | Open-ended questioning; reduced evaluation pressure | Non-evaluative responses and participation safety | Participation safety and bounded autonomy through teacher scaffolding |
| The situation was clearly defined, but we decided for ourselves how to express it. (SA04) | Clear task structure; expressive choice | Coexistence of structure and choice | Participation safety and bounded autonomy through teacher scaffolding |
| After several weeks, we knew what the other person was going to do without speaking. (SA05) | Movement continuation; nonverbal collaboration | Peer connection through shared activity | Peer connection and group belonging through embodied collaboration |
| We all did movements that looked rather silly, and that made me feel that I was part of the class. (SD02) | Shared embodied activity; group belonging | Belonging through collective participation | Peer connection and group belonging through embodied collaboration |
| It was not something I thought up; my body reacted on its own. (SA03) | Bodily response; role experience | Bodily experience and emotional resonance | Emotional resonance and self-awareness through embodied meaning-making |
| The emotion was too intense, as though the role had swallowed me. (SC06) | Intense emotion; difficulty exiting the role | Limitations of embodied experience | Bodily awareness, transfer, and risk (limiting evidence) |

***Supplementary Table S4. Final Themes and Representative Evidence across Multiple Data Sources***

| **Core theme** | **Subtheme** | **Interview data** | **Reflective journals** | **Classroom observations** | **Course documents** |
| --- | --- | --- | --- | --- | --- |
| Participation safety and bounded autonomy through teacher scaffolding | Non-evaluative guidance and participation safety | Accounts from SB02, SA03, SB04, and teaching staff | Descriptions of open-ended teacher responses and individualized attention | Teachers across all four settings used open-ended questions and reduced their direct intervention during later course stages | Course objectives emphasized participation, exploration, and multiple forms of expression |
|  | Structural scaffolding and bounded autonomy | Accounts from SA04, SA06, SB05, and teaching staff | Descriptions of safety provided by structure and uncertainty caused by unclear boundaries | Clearly defined tasks supported entry into activities, whereas excessive openness was sometimes accompanied by hesitation | Activity plans included scenarios, task requirements, and opportunities for open expression |
| Peer connection and group belonging through embodied collaboration | Nonverbal collaboration and the development of mutual trust | Accounts from SC05, SA05, SD06, and T04 | Descriptions of a progression from awkwardness to interpersonal coordination | Gaze, movement continuation, and nonverbal responses increased over time | Mirroring, paired improvisation, and collaborative activities |
|  | Peer acceptance and group belonging | Accounts from SD02, SB03, and SC05 | Descriptions of belonging within dyads, small groups, or the wider class | Self-directed collaboration and peer responses increased during the middle and later course stages | Group games and collective reflection emphasized shared participation |
| Emotional resonance and self-awareness through embodied meaning-making | Embodied empathy and emotional resonance | Accounts from SA03, SB03, SD01, and teaching staff | Descriptions of emotions elicited by bodily posture and role enactment | During later course stages, students more frequently articulated bodily and emotional experiences | Role-play and embodied expression constituted central course activities |
|  | Bodily awareness, transfer, and risk | Accounts from SC03, SC06, SC05, and T06 | Descriptions of bodily signals, experiences beyond the course, and emotional overload | Bodily awareness was observed alongside delayed role exit and avoidance | Some courses included reflection, relaxation, and de-roling activities |

***Supplementary Table S5. Matrix of Theme Manifestations across the Four Course Settings***

| **Theme manifestation** | **University A** | **University B** | **University C** | **University D** | **Shared pattern** |
| --- | --- | --- | --- | --- | --- |
| Participation safety | Developed through open-ended exploration | Developed through clearly defined situations | Developed through reflective responses | Developed through low-pressure games | Teachers rarely directly rejected or invalidated students’ expressions |
| Bounded autonomy | Greater scope for spontaneous expression | Choice exercised within a clearly defined framework | Opportunities to express different feelings and interpretations | Autonomous participation within the rules of group games | Autonomy depended on the coexistence of structure and choice |
| Peer connection | Movement-based coordination through improvisation and mirroring | Task continuation between role partners | Emotional responsiveness through psychodrama | Shared rhythms developed through group activities | Trust gradually developed through repeated collaboration |
| Group belonging | Primarily experienced within dyads and small groups | Primarily experienced among role partners and small groups | More frequently extended to the whole class | Whole-class belonging developed comparatively early | Having one’s expression acknowledged and extended was central to experiences of belonging |
| Embodied meaning | Emphasis on immediate bodily responses | Emphasis on role and situational immersion | Emphasis on post-activity meaning-making | Emphasis on bodily sensations arising from shared activity | Bodily experience required recognition and interpretation |
| Limiting experiences | Unclear boundaries and barriers to expression | Structure could restrict spontaneous expression | Emotional overload and insufficient time for reflection | Activities could remain at the level of surface participation | Activity formats could both enable and constrain learning experiences |

***Supplementary Table S6. Data Supporting the Recursive Participation Process***

| **Cross-theme relational stage** | **Primary data sources** | **Experiences represented in the data** | **Interrupting or limiting conditions** |
| --- | --- | --- | --- |
| Initial teacher scaffolding | SB02 and SA04; teaching-staff interviews, classroom observations, and course documents | Reduced concerns about evaluation and more comprehensible participation boundaries | Excessive direction, unclear boundaries, or mismatched task difficulty |
| Entry into shared embodied activity | SC05 and SD06; observation records from mirroring and improvisational activities | Mutual attention, movement continuation, and rhythmic coordination | Awkwardness, peer evaluation, or failure to respond |
| Peer connection and relational safety | SD02, SB03, and T04; reflective journals and classroom observations | Belonging within dyads, small groups, or the wider class, together with acknowledgment of individual expression | Group conflict, exclusion within established peer relationships, or insufficient attention |
| Bodily experience and meaning-making | SA03 and SC03; interviews, reflective journals, and collective-reflection records | Emotional resonance, self-awareness, and application of experience across situations | Insufficient reflection, excessive emotional intensity, or difficulty exiting a role |
| Experiences from earlier activities and subsequent participation | SB04; observations from the early, middle, and later course stages | More active participation, collaboration, and expression in subsequent activities | Pressure, perceived failure, or emotional overload accompanied by withdrawal |
